# Roux-En Y Gastric Bypass Results in Long-Term Remission of Hepatocyte Apoptosis and Hepatic Histological Features of Non-alcoholic Steatohepatitis

**DOI:** 10.3389/fphys.2016.00344

**Published:** 2016-08-19

**Authors:** Anne-Sophie Schneck, Rodolphe Anty, Stéphanie Patouraux, Stéphanie Bonnafous, Déborah Rousseau, Cynthia Lebeaupin, Beatrice Bailly-Maitre, Arnaud Sans, Albert Tran, Jean Gugenheim, Antonio Iannelli, Philippe Gual

**Affiliations:** ^1^Institut Nationale de la Santé et de Recherche Médicale, U1065, C3M, Team 8 “Hepatic Complications in obesity”Nice, France; ^2^Institut Nationale de la Santé et de Recherche Médicale, C3M, Université Côte d'AzurNice, France; ^3^Digestive Centre, Archet Hospital Nice, Centre Hospitalier Universitaire de NiceNice, France; ^4^Biological Centre, Pasteur Hospital, Centre Hospitalier Universitaire de NiceNice, France

**Keywords:** liver, NASH, bariatric surgery, NAFLD, steatosis, obesity

## Abstract

The long-term effects of bariatric surgery on non-alcoholic steatohepatitis (NASH), focusing on liver injury and hepatocyte apoptosis, are not well-established. We here performed a longitudinal study with paired liver biopsies of nine morbidly obese women (median BMI: 42 [38.7; 45.1] kg/m^2^) with NASH with a median follow-up of 55 [44; 75] months after laparoscopic Roux-en-Y gastric bypass (LRYGB) surgery. LRYGB surgery was associated with significant weight loss (median BMI loss −13.7 [−16.4; −9.5] kg/m^2^), improved hepatic steatosis in all patients (55.5% with total resolution), and resolution of hepatic inflammation and hepatocyte ballooning in 100 and 88.8% of cases, respectively. Alanine aminotransferase levels dropped to normal values while hepatic activated cleaved caspase-3 levels strongly decreased after a median follow-up of 55 months. Hepatocyte apoptosis, as evaluated by serum caspase-generated keratin-18 fragment, improved within the first year following LRYGB and these improvements persisted for at least 55 months. LRYGB in morbidly obese patients with NASH is thus associated with a long-lasting beneficial impact on hepatic steatohepatitis and hepatocyte death.

## Introduction

Obesity represents a major health burden, as it is associated with a growing number of comorbidities (Must et al., 1999; Stevens et al., 2012). As the prevalence of obesity increases, so does the prevalence of non-alcoholic fatty liver disease (NAFLD), which is now the leading cause of chronic liver disease in the Western world (Younossi et al., 2011, 2016; Setiawan et al., 2016). NAFLD includes a spectrum of liver abnormalities, ranging from simple steatosis to non-alcoholic steatohepatitis (NASH) and to liver cirrhosis, eventually leading to hepatocellular carcinoma (Tran and Gual, 2013; Yeh and Brunt, 2014). Regarding NASH, which is characterized by the presence of liver inflammation and injury (hepatocyte ballooning and apoptosis), recent reports indicate that it is the second most common indication for liver transplantation in the United States (Wong et al., 2015). To counter the obesity epidemic, bariatric surgery has emerged as the only therapeutic treatment that results in long-term weight loss and improvement or resolution of most obesity-related comorbidities (Mitka, 2012; Schauer et al., 2014). However, evidence of the long-term efficacy of bariatric surgery against NASH is not well-established. A recent report, including a cohort of 109 morbidly obese patients with biopsy-proven NASH, has shown that bariatric surgery induced the disappearance of NASH in nearly 85% of patients and reduced the pathological features of the disease after 12 months of follow-up (Lassailly et al., 2015). Other results reported in the literature are more disparate and focus on various liver complications (mainly steatosis and fibrosis) with differing bariatric procedures and with a follow-up never exceeding 24 months (Silverman et al., 1995; Clark et al., 2005; Mattar et al., 2005; Mottin et al., 2005; Barker et al., 2006; Csendes et al., 2006; de Almeida et al., 2006; Klein et al., 2006; Furuya et al., 2007; Liu et al., 2007; Mathurin et al., 2009; Chavez-Tapia et al., 2010). In this study, we evaluated the long-term effects (median follow-up biopsy at 55 months) of laparoscopic Roux-en-Y gastric bypass (LRYGB) surgery on hepatic NASH features and liver injury (mainly hepatocyte apoptosis) in morbidly obese women with biopsy-proven NASH at the time of the surgery.

## Materials and methods

### Study design

Five hundred and sixty-eight consecutive severely and morbidly obese patients, referred for bariatric surgery, were included between December 2002 and December 2009. The study protocol was performed according to the French legislation regarding Ethics and Human Research and was approved by the local Ethics Committee (Huriet-Serusclat law, DGS 2003/0395). Written informed consent was obtained from all patients. All patients met the 1992 NIH Consensus Conference guidelines for gastrointestinal surgery for obesity. These patients underwent bariatric surgery at the Department of Digestive Surgery and Liver Transplantation of the University of Nice (France). All patients had a preoperative work-up (repeated at 6 and 12 months after surgery) and underwent a wedge-liver biopsy at the time of surgery. A second liver biopsy and a concomitant diagnostic work-up were offered to patients who underwent a LRYGB, and who initially presented with criteria for NASH (on a liver biopsy) and completed a minimum follow-up period of 40 months after surgery. Between January 2002 and December 2009, of the 84 patients with a NAS ≥ 5 on the wedge-liver biopsy at the time of surgery, 33 had a follow-up of <36 months, 13 were lost to follow-up and 29 refused to reiterate the biopsy. The comparison of clinical and biochemical parameters of the nine NASH patients with a second liver biopsy (Table 1) compared with those corresponding to the 75 NASH patients without a second liver biopsy (Table 2) showed no significant differences regarding BMI (*P* = 0.2473), fasting glucose level (*P* = 0.177), fasting insulin level (*P* = 0.311), HOMA-IR (*P* = 0.5976), prevalence of type 2 diabetes (44 vs. 42.6% respectively), ALT level (*P* = 0.4038) and CRP level (*P* = 0.1828). Overall, nine women met all the criteria and accepted the second biopsy. In addition, data from blood samples obtained from five morbidly obese patients, included in the same prospective ongoing study and who had no sign of NAFLD on liver histology (aged 37 ± 10 years; BMI 44 ± 3 kg/m^2^), from seven patients with biopsy-proven severe hepatic steatosis (aged 34 ± 8 years; BMI 46 ± 8 kg/m^2^), and from seven patients with biopsy-proven NASH (aged 40 ± 8 years; BMI 41 ± 3 kg/m^2^) were used in this study.

**Table 1:** **Patients' clinical and biochemical parameters at baseline and after a ≥ 40-month follow-up period (patients with paired liver biopsies)**.

	**T0**	***T* ≥ 40**	***p***
Number	9	9	ns
Time after surgery (months) (median, Q1; Q3)		55 (44; 75)	
BMI (kg/m^2^) (median, Q1; Q3)	42.0 (38.7; 45.1)	27.1 (24.3; 31.8)	0.042
Δ BMI (kg/m^2^) (median, Q1; Q3)		−13.7 (−16.4; −9.5)	
Fasting glucose (mmol/l) (median, Q1; Q3)	7.3 (5.3; 9.0)	4.7 (3.8; 5.5)	0.031
Fasting insulin (mUI/l) (median, Q1; Q3)	24.7 (18.0; 33.0)	11.8 (6.5; 19.2)	0.032
HOMA-IR (median, Q1; Q3)	7.4 (3.5; 10.8)	1.9 (1.3; 3.5)	0.008
Diabetes (%)	4 (44)	0 (0)	ns
Metabolic syndrome (%)	7 (77)	2 (22)	ns
CRP (mg/dl) (median, Q1; Q3)	7.7 (5.7; 9.6)	0.6 (0.4; 2.4)	0.004

*T0, baseline; T ≥ 40, after a ≥ 40-month follow-up period. BMI, Body-mass index; Δ BMI, Excess BMI loss; HOMA-IR, Homeostasis Model Assessment of Insulin Resistance; CRP, C-reactive protein; ns, not significant; p, the Mann-Whitney test or Fisher's exact test*.

**Table 2:** **Patients' clinical and biochemical parameters at baseline and after a 12-month follow-up period (patients without a second liver biopsy)**.

	**T0**	**T0**	**T12**	***p***
Number	75	30	30	
Gender (F/M)	57/18	23/7		
BMI (kg/m2) (median, Q1; Q3)	43.7 (40.2; 47.2)	45.5 (41.0; 48.6)	28.8 (25.5; 32.3)	<0.001
Δ BMI (kg/m2) (median, Q1; Q3)			−15.1 (−19.9; −13.4)	
Fasting glucose (mmol/l) (median, Q1; Q3)	5.5 (5.1; 7.4)	5.8 (5.1; 9.7)	4.7 (4.1; 5.2)	<0.001
Fasting insulin (mUI/l) (median, Q1; Q3)	28.0 (18.0; 44.0)	27.0 (17.2; 39.5)	6.8 (4.9; 12.0)	<0.001
HOMA-IR (median, Q1; Q3)	7.8 (4.8; 12.4)	7.6 (5.8; 11.3)	1.3 (1.0; 2.1)	<0.001
Diabetes (%)	32 (42.6)	18(60)	4 (13.3)	<0.001
Metabolic syndrome (%)	36 (48)	21 (70)	9 (30)	0.002
CRP (mg/dl) (median, Q1; Q3)	9.0 (6.0; 14.5)	9.0 (6.8; 14.5)	2.9 (1.1; 4.1)	<0.001
ALT (IU/L) (median, Q1; Q3)	50.5 (36.0; 72.5)	53.5 (35.7; 69.5)	24.0 (18.0; 32.0)	<0.001
NAS (grade) (median, Q1; Q3)	5.0 (5.0; 5.0)	5.0 (5.0; 5.0)		

*T0, baseline; T12, 12-month follow-up. BMI, Body-mass index; Δ BMI, Excess BMI loss; HOMA-IR, Homeostasis Model Assessment of Insulin Resistance; CRP, C-reactive protein; ns, not significant; p, the Mann-Whitney test or Fisher's exact test*.

### Population characteristics

All patients were negative for hepatitis B and C viral markers, auto-antibodies indicative of autoimmune hepatitis, and had self-reported negligible alcohol consumption (<20 g/day in women and <30 g/day in men). Alcohol abuse was also excluded by interviewing the patients' relatives. Patients with a history of inflammatory disease (including rheumatoid arthritis, systemic lupus erythematosus, and inflammatory bowel disease), current infections, recent history of cancer (<5 years), and severe pulmonary or cardiac disease were not enrolled in the study. The patients' characteristics are described in Table 1. Before surgery and during the follow-up (6, 12 months and the last follow-up visit before the second liver biopsy), fasting blood samples were obtained and analyzed for alanine aminotransferase (ALT), glucose and insulin, triglycerides, high-density lipoprotein (HDL)-cholesterol, C-reactive protein, and caspase-generated keratin (K18 fragment). Metabolic syndrome was defined according to the modified International Diabetes Federation (IDF) criteria of three or more of the following: (i) central obesity defined by an increased waist circumference (≥80 cm), (ii) triglycerides ≥1.7 mmol/L or treatment for hyper-triglyceridemia; (iii) HDL-cholesterol <1.29 mmol/L; (iv) systolic blood pressure ≥130 mmHg or diastolic blood pressure ≥85 mmHg or treatment for hypertension, and (v) fasting plasma glucose ≥5.6 mmol/L or previously diagnosed type-2 diabetes mellitus (Alberti et al., 2006). Type-2 diabetes was defined by two measurements of elevated fasting plasma glucose ≥7 mmol/L. Insulin resistance was evaluated using the homeostatic model assessment (HOMA-IR) index (Wallace et al., 2004). Nine patients (only women) with biopsy-proven NASH had liver biopsies repeated at a median follow-up of 55 [44; 75] months after surgery. The first liver biopsy was a wedge biopsy obtained at the beginning of the LRYGB, with no ischemic preconditioning. The second was a needle-biopsy of the liver, obtained using the percutaneous approach. The quality of the biopsies of the nine included patients was sufficient for interpretation and the length of each liver biopsy was over 15 mm. It is however important to underline the fact that potential limitations of comparing wedge to needle biopsies exist. Biopsies were stained with hematoxylin–eosin–saffron and sirius red. Liver biopsies were reviewed by two liver pathologists who were blinded to the clinical and biological characteristics of the patients. Histopathological analyses were performed according to the scoring system of Kleiner et al. (2005). Four histopathological features were semi-quantitatively evaluated: grade of steatosis (0, <5%; 1, 5–33.3%; 2, >33.3–66.6%; 3, >66.6%), lobular inflammation (0, no inflammatory foci; 1, <2 inflammatory foci per 200x field; 2, 2–4 inflammatory foci per 200x field; 3, >4 inflammatory foci per 200x field), hepatocellular ballooning (0, none; 1, few balloon cells; 2, many cells/prominent ballooning), and liver bridging fibrosis (classified into seven stages according to the NASH Clinical Research Network Scoring System Definition F0, no fibrosis; F1a, mild zone 3 sinusoidal fibrosis; F1b, moderated zone 3 sinusoidal fibrosis; F1c, peri-portal sinusoidal fibrosis; F2, zone 3 sinusoidal fibrosis and peri-portal sinusoidal fibrosis; F3, bridging fibrosis; and F4, cirrhosis). The NAFLD activity score (NAS) is defined as the unweighted sum of scores for steatosis (0–3), lobular inflammation (0–3), and ballooning (0–2), thus ranging from 0 to 8 (Kleiner et al., 2005).

### Circulating levels of transaminases and K18 fragment

Plasma alanine aminotransferase (ALT) levels were determined using an *in vitro* test with pyridoxal phosphate activation on a Roche/Hitachi cobas c system (ALTPM, cobas, Meylan, France). Keratin 18 (K18) is cleaved by the caspases during apoptosis, generating soluble protein fragments. The M30 Apoptosense® ELISA assay specifically measures apoptosis (the caspase-generated K18 fragment, K18-Asp396). All samples were analyzed in duplicate following the manufacturer's instructions. The within assay (WA% CV) variation was <10% and between assay (BA% CV) variation was <10% for samples >100 U/L. The minimum detectable concentration was 25 U/L. Keratins are released into the circulation as protein complexes. These complexes are remarkably stable during sample collection and long-term storage. Furthermore, plasma/serum samples can be exposed to repetitive freeze–thaw cycles without loss of activity (Olofsson et al., 2007).

### IHC analysis

Immunostaining for cleaved caspase-3 (Asp175) was performed using rabbit plolyclonal antibodies against amino-terminal residues adjacent to (Asp175) in human caspase 3. Sections measuring 2 μm were cut from each paraffin block and were put to dry at 37°C during 12 h. After deparaffinization and rehydration, all sections were pretreated at pH 6 with Flex TRS Low (PTLink DAKO, Glostrup, Denmark) during 20 min. Endogenous peroxidase was blocked in 1% hydrogen peroxide for 5 min (DAKO, Glostrup, Denmark) at room temperature. After rinsing with phosphate buffered saline, the sections were incubated with cleaved caspase-3 (Asp175) antibody (#9661, Cell Signaling) for 20 min at room temperature. Then sections were incubated with an appropriate secondary antibody from the Envision flex/HRP kit (Dako, Glostrup, Denmark) for 20 min, at room temperature. Next, slides were incubated in PBS, for 20 min, at room temperature, and then peroxidase activity was detected by diaminobenzidinetetrahydrochloride for 8 min and used for visualization and haematoxylin (Dako, Glostrup, Denmark) during 6 min for nuclear counterstaining.

### Statistical analyses

The statistical significances between the two study groups were determined using the non-parametric Mann-Whitney test and Fischer's test. A *P* < 0.05 was considered statistically significant. Quantitative variables are presented as their medians (interquartile ranges).

## Results

The aim of this study was to investigate the potentially long-lasting beneficial effects of LRYGB on obesity-related liver complications. To this end, 84 morbidly patients with biopsy-proven NASH diagnosed at time of bariatric surgery (LRYGB) were studied. Of these, nine women with a median age of 51 [35; 59] years at the time of LRYGB had a second liver biopsy after ≥40 months of follow-up (Table 1).

**Figure 1:**
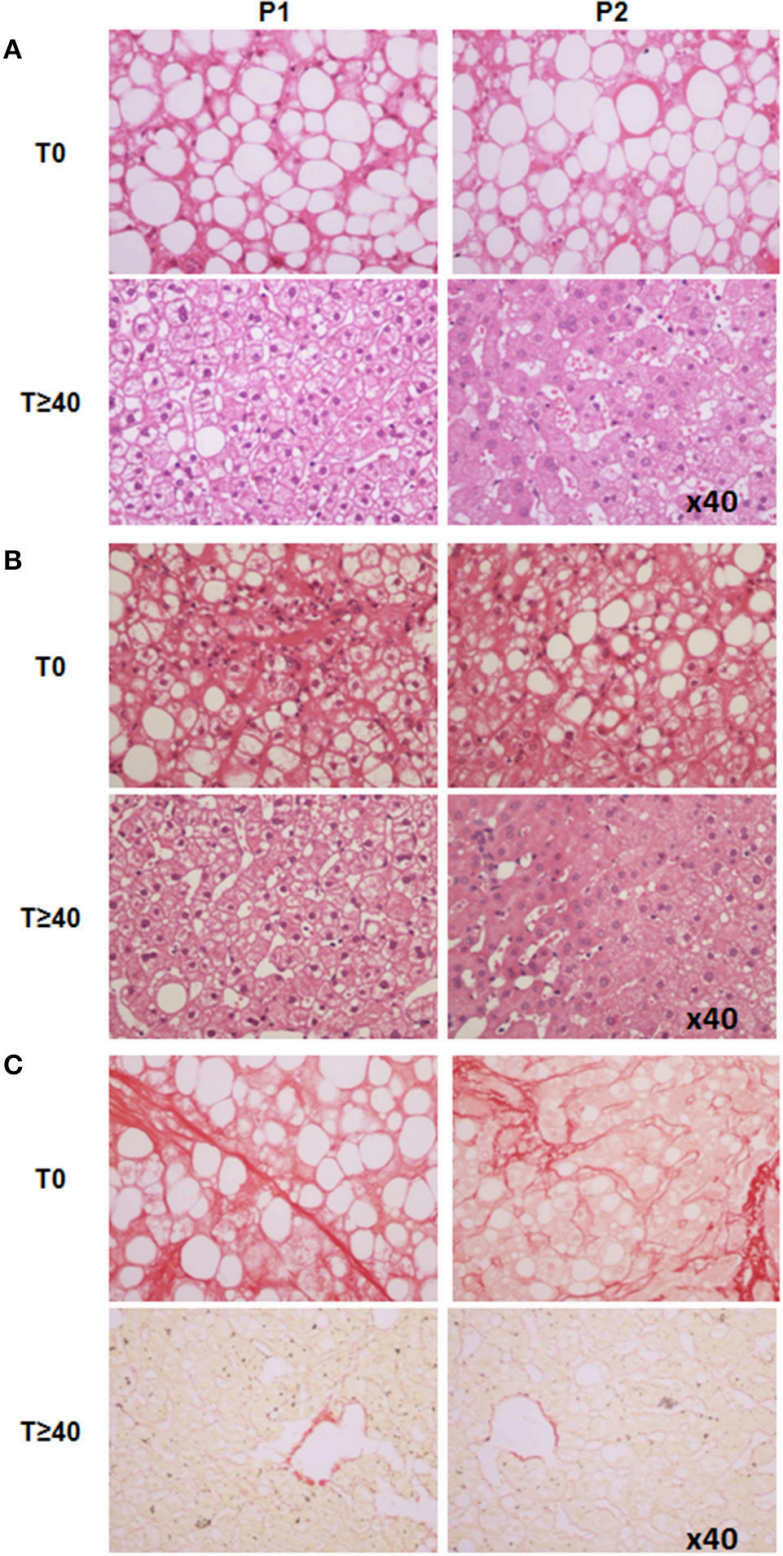
**Liver histology analysis of two representative patients (P1 and P2) at baseline and ≥40-months after LRYGB. (A)** Liver steatosis was improved (HES staining, x40). **(B)** Ballooned hepatocytes and hepatic inflammation, both present at baseline, were no longer present on the second liver biopsy (HES staining, x40). **(C)** P1 had bridging fibrosis (*F* = 3), P2 had zone three sinusoidal fibrosis and peri-portal sinusoidal fibrosis (*F* = 2). The second liver biopsy showed a significant improvement in fibrosis (*F* = 0) in both patients (Sirius red straining, x40). T0, baseline; T ≥ 40, after a ≥40-month follow-up period.

### LRYGB improves the metabolic syndrome, type-2 diabetes, and systemic inflammation

We first evaluated the effects of LRYGB on weight loss, the metabolic syndrome, type-2 diabetes and systemic inflammation in our NASH patients with paired-liver biopsies. All patients lost more than 50% of excess BMI and had a median loss of −13.7 [−16.4; −9.5] kg/m^2^ BMI points (Table 1). Insulin resistance, as evaluated by the HOMA-IR, fasting insulin and glycaemia were strongly improved after LRYGB, as shown in Table 1. Four patients with type-2 diabetes before surgery were in remission at the time of the follow-up. The metabolic syndrome was diagnosed in seven patients at the time of the initial surgery and persisted in only two patients by the end of the follow-up. Chronic low-grade inflammation, as evaluated by C-reactive protein, was also improved after LRYGB in all patients (Table 1). The beneficial effect of LRYGB on the metabolic syndrome and systemic inflammation could already occur 1 year after bariatric surgery as previously reported (Mathurin et al., 2006; Anty et al., 2008; Bertola et al., 2009). In line with this, the analysis of the clinical and biochemical parameters at baseline and at 12 months after LRYGB of 30 out of our 75 patients without second liver biopsy also showed a significant improvement of fasting insulin, glycaemia, HOMA-IR, metabolic syndrome, diabetes, and CRP (Table 2).

### LRYGB improves hepatic steatosis, inflammation, and NAFLD activity score (NAS) in all patients, and improves hepatic fibrosis in a large majority

**Figure 2:**
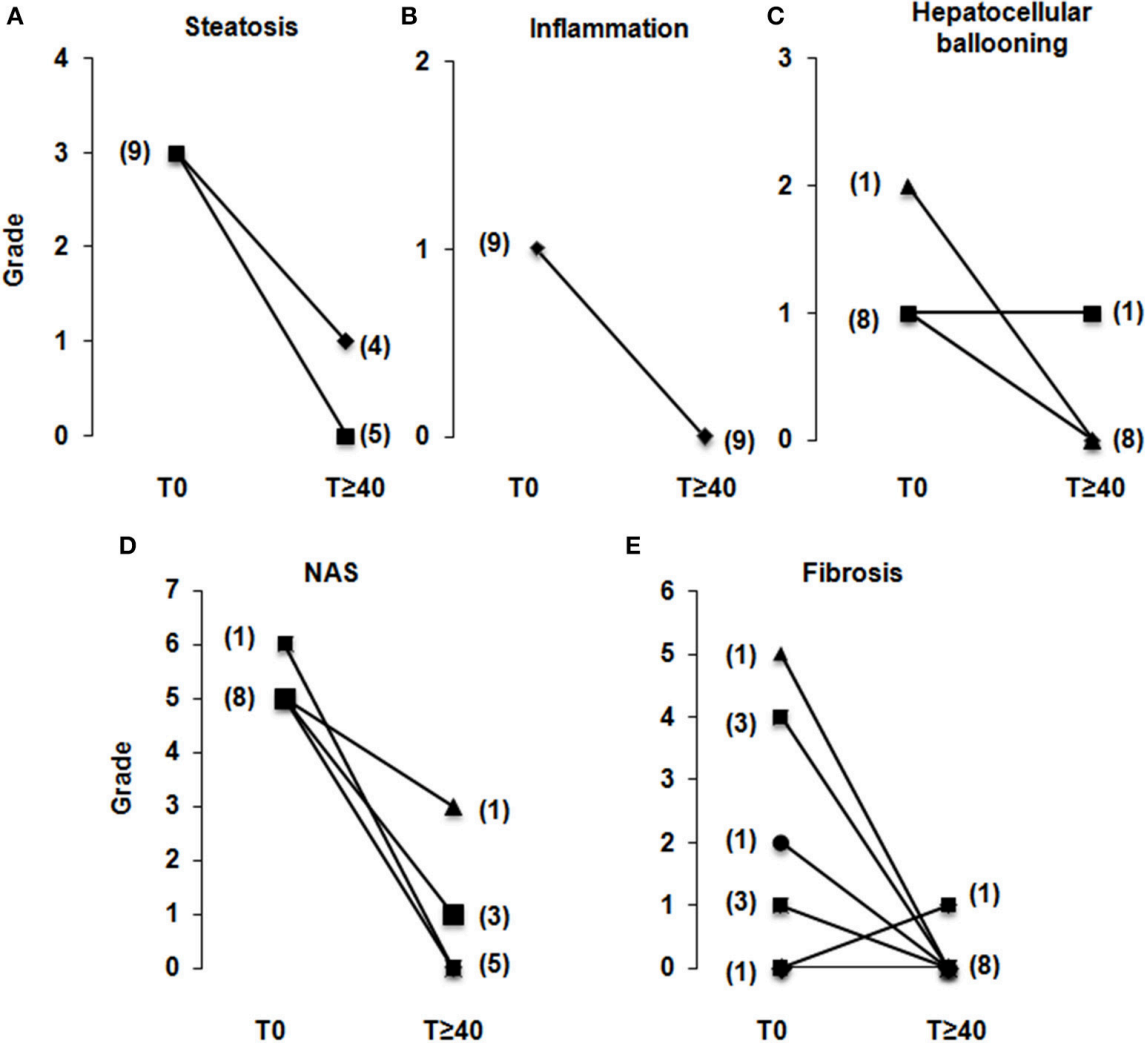
**LRYGB in NASH patients improves hepatic steatosis and inflammation in all patients, and improves hepatic fibrosis in the large majority after a median follow-up of 55 months**. Nine morbidly obese patients with biopsy-proven NASH underwent LRYGB and had a second liver biopsy at a median follow-up period of 55 [44; 75] months after surgery. From the paired liver biopsies, steatosis **(A)**, inflammatory foci **(B)**, hepatocellular ballooning **(C)**, and fibrosis **(E)** were evaluated. **(D)** The NAFLD activity score (NAS) was evaluated as described in Section Materials and Methods. Fibrosis was semi-quantitatively evaluated as follows: 0, none; 1, perisinusoidal or periportal mild (1A); 2, moderate (1B); 3, portal/periportal (1C); 4, perisinusoidal and portal/periportal; 5, bridging fibrosis; 6, cirrhosis. (*N*) = number of patients.

We then evaluated the effects of LRYGB on obesity-related liver complications. Liver steatosis was evaluated as severe (S3, >66.6% of hepatocytes) in all patients at the time of surgery. On the second biopsy, steatosis was improved in all patients: i.e., full correction in five patients (55.5%) and grade 1 (44.4 %) in four patients (Figures 1A, 2A). The correction of hepatic steatosis was associated with a more important loss of weight as evaluated by percentage of initial body weight (39 ± 6 vs. 24 ± 3%, *P* = 0.02). Hepatic inflammation, present in all patients at the time of the surgery, was no longer present on the second biopsy in any patient (Figures 1B, 2B). Ballooned hepatocytes, another hallmark of NASH and a marker of liver-cell degeneration, were found in all liver biopsies at the time of surgery, but were no longer present in the second liver biopsy in eight patients (88.8%; Figures 1B, 2C). Only one patient still had ballooned hepatocytes on the liver biopsy in spite of significant weight loss, improved metabolic syndrome, insulin resistance (HOMA-IR: from 5.4 to 2.3), hepatic steatosis (from S3 to S1) and hepatic inflammation (Figure 2C). As a consequence, the NAS, which was elevated in all patients at the time of surgery (eight patients with NAS = 5, and one with NAS = 6), dropped considerably in all patients by 2–6 full points (Figure 2D). The stage of fibrosis was more heterogeneous at the time of surgery, with only one patient showing advanced fibrosis (*F* = 3), three patients with moderate fibrosis (*F* = 2), four patients with mild fibrosis (1 with *F* = 1B, 3 with *F* = 1A) and one patient with no fibrosis (*F* = 0). The second liver biopsy showed a significant improvement in fibrosis (*F* = 0) in seven patients and a slight progression of liver fibrosis in one patient (from F0 to F1A; Figures 1C, 2E). One patient without fibrosis at the time of the surgery showed no signs of fibrosis on the second biopsy. LRYGB was associated with corrected hepatic steatosis and inflammation in all patients, and improvement of fibrosis in 88.8% of patients.

### LRYGB improves hepatic injury and hepatocyte apoptosis in all patients

**Figure 3:**
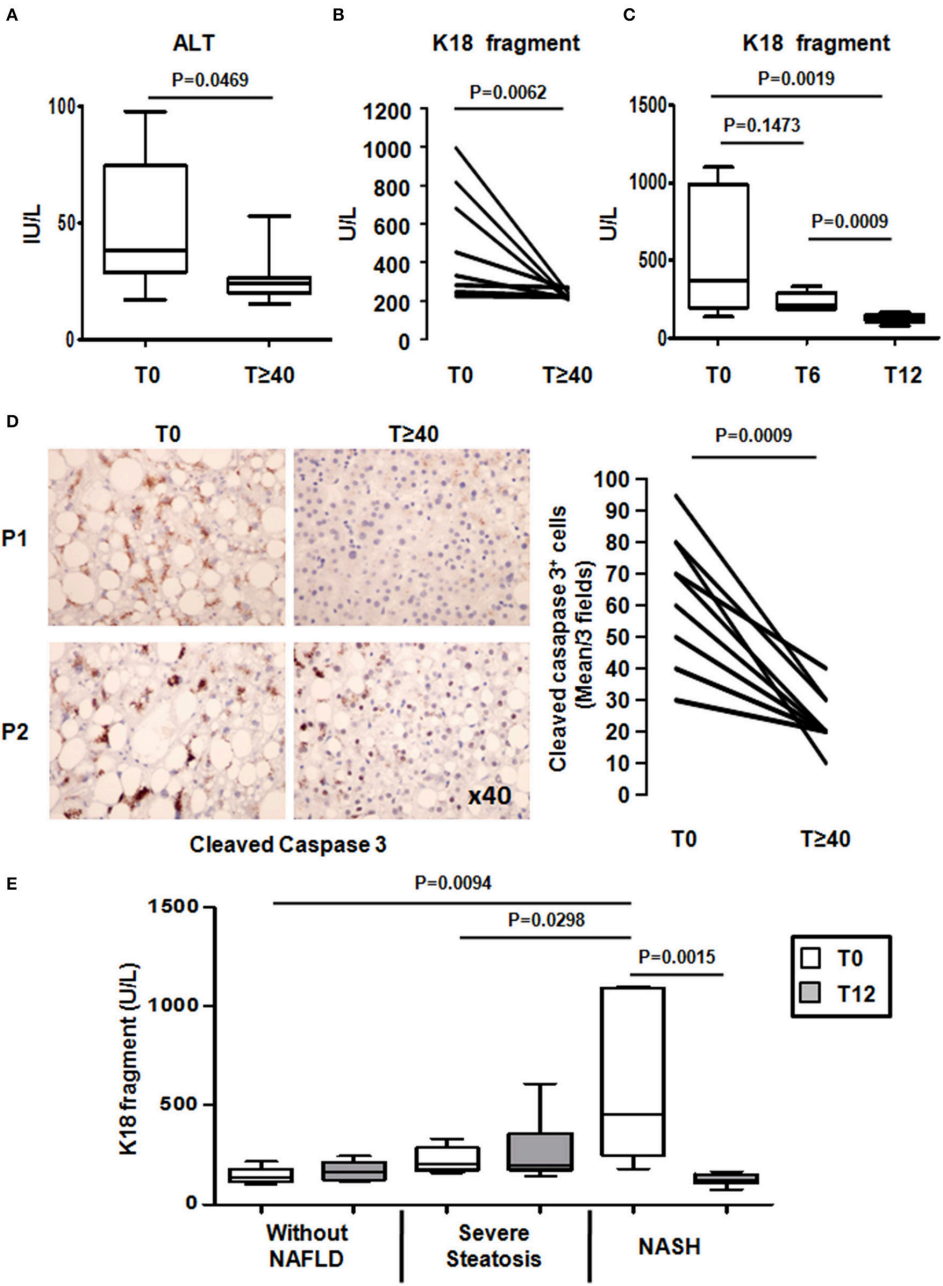
**LRYGB improves liver injury and hepatocyte apoptosis in NASH patients after a median follow-up of 55 months**. Serum levels of **(A)** alanine aminotransferase (ALT) and **(B)** a marker of hepatocyte apoptosis [caspase-generated keratin 18 fragment (K18 fragment)] and **(D)** hepatic levels of cleaved caspase 3 were evaluated at baseline and at the median follow-up of 55 [44; 75] months after LRYGB in NASH patients. The levels of K18 fragment were also evaluated **(C)** at 6 months and at 1 year after a LRYGB and, **(E)** at baseline (T0) and 1 year (T12) after LRYGB in three additional groups of patients without NAFLD (*n* = 5), severe steatosis (*n* = 7), or NASH (*n* = 7). Results are expressed as the median [25th, 75th percentiles] **(A,C,E)**.

NASH is also characterized by the substantial death of hepatocytes (Feldstein and Gores, 2005; Tran and Gual, 2013). Hepatocyte apoptosis plays an important role in the progression and the severity of obesity-related liver complications. We thus investigated the long-term effect of LRYGB on hepatocyte injury. ALT serum levels reached normal-range values in all patients by the last follow-up (ALT: 38 [29; 74] to 24 [20; 26] UI/L; Figure 3A). The serum levels of caspase-generated keratin 18 fragment (K18 fragment) were used to evaluate hepatocyte apoptosis. An average decrease of 35% in serum K18 fragment levels were found in 88.8% of patients after a median follow-up of 55 [44; 75] months after LRYGB (Figure 3B). One patient who already had low serum K18 fragment levels at the time of the LRYGB showed no significant changes (Figure 3B). Interestingly in the initially NASH patients, the K18 fragment levels reached those usually found in patients without hepatic complications just 1 year after LRYGB (Anty et al., 2010; Lavallard et al., 2011; Figure 3C). We then determined the hepatic caspase 3 activity, as evaluated by the cleaved caspase 3 (Asp175) levels in the paired liver biopsies with a median follow-up of 55 [44; 75] months after LRYGB. As shown in Figure 3D, the percentage of cleaved caspase 3 positive hepatocytes strongly decreased (−40% average decrease) in all patients at the time of follow-up. We also evaluated the K18 fragment levels in three additional groups of morbidly obese patients without any signs of NAFLD (*n* = 5), severe steatosis (*n* = 7), or severe steatosis associated with NASH (*n* = 7): assessed from a liver biopsy at baseline and at 1 year after LRYGB. While the K18 fragment levels showed no significant difference in patients with hepatic steatosis, they were increased in patients with NASH compared with patients without NASH at the time of surgery (Figure 3E). At 1 year after LRYGB, the levels of the K18 fragment had strongly decreased in all NASH patients (Figure 3E). Altogether, these data indicate that LRYGB had a beneficial effect on hepatocyte apoptosis by 1 year post-surgery, and that this was maintained for the median follow-up period of 55 [44; 75] months.

## Discussion

While there is strong evidence for the beneficial effects of the LRYGB on excess weight and resolution or reduction of type 2-diabetes (with remission in 63.5% of cases; Ribaric et al., 2014), its long term impact on liver histology in NASH patients needs to be better characterized. Most studies with paired liver biopsies reported a mean interval between LRYGB and a second liver biopsy of 19 ± 4 (range: 12–25) months (Silverman et al., 1995; Clark et al., 2005; Mattar et al., 2005; Mottin et al., 2005; Barker et al., 2006; Csendes et al., 2006; Klein et al., 2006; Furuya et al., 2007; Liu et al., 2007; Lassailly et al., 2013).

In this study, a second biopsy was performed on previously morbidly obese patients with liver biopsy-proven severe steatosis and NASH after a median interval of 55 [44; 75] months after LRYGB. As reported herein and previously, 1 year after surgery (Anty et al., 2008; Bertola et al., 2009; Ribaric et al., 2014; Table 2), insulin resistance, the metabolic syndrome and systemic inflammation had all improved. These positive effects are thus maintained after a median follow-up period of 55 [44; 75] months. A beneficial impact of LRYGB on hepatic steatosis was also observed in all of our patients, from total resolution (in 55.6% of cases) to striking improvement (S3 to S1: 44.4%). As previously reported, LRYGB already improved hepatic steatosis, evaluated by paired liver biopsies, at a median follow up of 18 [12; 23] months after LRYGB (Silverman et al., 1995; Clark et al., 2005; Mattar et al., 2005; Mottin et al., 2005; Barker et al., 2006; Klein et al., 2006; Furuya et al., 2007; Liu et al., 2007). Furthermore, the improvement of hepatic steatosis is not specific to a LRYGB procedure. A recent meta-analysis compiled results from different bariatric procedures, including LRYGB, gastric banding, sleeve gastrectomy, duodenal switch, and biliopancreatic diversion and reported an improvement of hepatic steatosis in 90% of cases (Chavez-Tapia et al., 2010). Therefore, our study demonstrates that the beneficial impact of LRYGB on insulin resistance, systemic inflammation, and hepatic steatosis obtained 1 year after surgery is maintained for at least 40 months (median 55 [44; 75]).

Regarding the hepatic inflammation and fibrosis, some studies with paired liver biopsies reported an improvement in the histopathological criteria for NASH in the short term (mean follow-up of 21.35 ± 4.5 months; Clark et al., 2005; Barker et al., 2006; de Almeida et al., 2006) and fibrosis (Clark et al., 2005; Mattar et al., 2005; Barker et al., 2006; Furuya et al., 2007) after LRYGB surgery. In our study, a median follow-up period of 55 months after LRYGB exhibited beneficial effects on inflammatory foci, ballooning and fibrosis. Our NASH patients showed 100% improvement in inflammatory foci and 88.8% improvement in ballooned hepatocytes after LRYGB surgery. As a consequence, the NAS decreased in 100% of cases. Despite the heterogeneous nature of the degree of hepatic fibrosis in our patients at the time of LRYGB surgery, hepatic fibrosis improved in 88.8% of cases. One patient showed a slight increase in fibrosis (from F0 to F1A). No explanation could be found concerning the patient with no resolution of hepatocyte ballooning. Indeed, this patient lost significant weight and had decreased metabolic syndrome features, decreased insulin resistance (HOMA-IR: from 5.4 to 2.3) and improved ALT levels. Other liver complications were also reduced, including hepatic steatosis (from S3 to S1), inflammation and fibrosis (from F2 to F0). A recent report has shown that the disappearance of NASH in nearly 85% of cases and decreased pathological features of NAFLD already occur 1 year after the bariatric surgery in a cohort of 109 morbidly obese patients with biopsy-proven NASH (Lassailly et al., 2015). The beneficial effect of bariatric surgery (mainly LRYGB procedure) on NASH could thus rapidly occur in the first year and be maintained up to 40 months after the surgery.

We next found that hepatocyte apoptosis, as evaluated by the serum K18 fragment had already improved 1 year after LRYGB and remained low until the last follow-up (at 55 [44; 75] months). This was also confirmed by a strong decrease in hepatic caspase 3 activity, as evaluated by the levels of hepatocyte cleaved caspase 3 for at least 40 months after LRYGB surgery. In our patients, this serum hepatocyte apoptotic marker increased approximately four-fold in patients with NASH, which is in accordance with previous reports on overweight, obese and severely obese patients (Wieckowska et al., 2006; Anty et al., 2008; Younossi et al., 2008; Feldstein et al., 2009; Tamimi et al., 2011; Joka et al., 2012; Shen et al., 2012), and correlates with NAS (*r* = 0.549, *P* < 0.001, *n* = 41). However, it has been recently reported that K18 fragment level could be inadequate as a screening test for staging NASH according to its limited sensitivity (Cusi et al., 2014). In concert, these data suggest that this non-invasive marker combined with other clinical/laboratory parameters may be helpful to monitor the evolution of NASH after bariatric surgery. Wai-Sun Wong et al. recently reported that the levels of the serum K18 fragment reflected disease activity in a prospective longitudinal study on overweight/obese patients undergoing paired liver biopsies with a follow-up time of 3 years (Wong et al., 2010).

The improvement in hepatocyte death and reduction of inflammation after LRYGB surgery could prevent the progression of hepatic complications. Indeed, apoptotic hepatocytes are engulfed by Kupffer cells, which results in activation and inflammation. The activation of stellate cells by apoptotic bodies or by TGFβ from activated Kupffer cells then leads to liver fibrosis (Malhi and Gores, 2008). Furthermore, a pan-caspase inhibitor or an overexpression of the anti-apoptotic Bcl2 protein was shown to reduce fibrosis in an animal model of NAFLD and fibrosis, respectively (Mitchell et al., 2009; Witek et al., 2009).

Although, the main weakness of the present study lies in the small size of the sample and the gender bias (only females were included), the exhaustive preoperative and postoperative work-up and the paired liver biopsies allowed for a complete characterization of our patients with NASH. We were thus able to demonstrate that the LRYGB surgery results in the concomitant remission of systemic inflammation, insulin resistance and NASH features (steatosis, inflammation, and hepatocellular ballooning) in all patients at a median follow-up of 55 months. We also found a rapid decrease in hepatocyte apoptosis, as evaluated by serum levels of K18 fragment. These results should be confirmed in additional studies with a larger sample size and a longer follow-up (>6 years) to better understand the molecular mechanisms that are involved in the remission of obesity-related liver complications after LRYGB surgery, as well as after other bariatric procedures to determine if they could share these beneficial effects. Because pharmacological therapy has only marginal and perhaps clinically irrelevant effects on NASH and fibrosis (Ratziu, 2013), and in light of our results, the implications for the protective effects of LRYGB surgery against the progression of obesity-related liver complications may become particularly relevant.

## Author contributions

AS, RA, AI, and PG: study concept and design; AS, RA, and SP: acquisition of data; AS, SP, SB, DR, AI, and PG: analysis and interpretation of data; AS, AI, and PG: drafting and critical revision of the article for important intellectual content; AS, CL, BB, AT, JG, AS, AI, and PG: precious help with editing the manuscript at different stages; RA, PG: statistical analysis; PG obtained funding; PG and AI: study supervision.

## Funding

This work was supported by grants from Inserm (France), the University of Nice, the Programme Hospitalier de Recherche Clinique (Centre Hospitalier Universitaire of Nice), and charities (Association Française pour l'Etude du Foie (AFEF)/LFB to PG, AFEF/Aptalis to BB, Société Francophone du Diabète (SFD) to PG, SFD/Roche Pharma to PG, SFD/MSD to BB). This work was also funded by the French Government (National Research Agency, ANR) through the “Investments for the Future” LABEX SIGNALIFE, program reference #ANR-11-LABX-0028-01 and #ANR-15-CE14-0016-01.

### Conflict of interest statement

The authors declare that the research was conducted in the absence of any commercial or financial relationships that could be construed as a potential conflict of interest.
